# Automatic measurement of exophthalmos based orbital CT images using deep learning

**DOI:** 10.3389/fcell.2023.1135959

**Published:** 2023-02-24

**Authors:** Yinghuai Zhang, Jing Rao, Xingyang Wu, Yongjin Zhou, Guiqin Liu, Hua Zhang

**Affiliations:** ^1^ School of Biomedical Engineering, Health Science Center, Shenzhen University, Shenzhen, China; ^2^ Marshall Laboratory of Biomedical Engineering, Shenzhen, China; ^3^ Shenzhen Eye Hospital, Jinan University, Shenzhen, China; ^4^ Shenzhen Eye Institute, Shenzhen, China; ^5^ Shenzhen Overseas Chinese Town Hospital, Shenzhen, China

**Keywords:** CT images, deep learning, exophthalmos, orbital diseases, thyroid-associated ophthalmopathy

## Abstract

**Introduction:** Objective, accurate, and efficient measurement of exophthalmos is imperative for diagnosing orbital diseases that cause abnormal degrees of exophthalmos (such as thyroid-related eye diseases) and for quantifying treatment effects.

**Methods:** To address the limitations of existing clinical methods for measuring exophthalmos, such as poor reproducibility, low reliability, and subjectivity, we propose a method that uses deep learning and image processing techniques to measure the exophthalmos. The proposed method calculates two vertical distances; the distance from the apex of the anterior surface of the cornea to the highest protrusion point of the outer edge of the orbit in axial CT images and the distance from the apex of the anterior surface of the cornea to the highest protrusion point of the upper and lower outer edges of the orbit in sagittal CT images.

**Results:** Based on the dataset used, the results of the present method are in good agreement with those measured manually by clinicians, achieving a concordance correlation coefficient (CCC) of 0.9895 and an intraclass correlation coefficient (ICC) of 0.9698 on axial CT images while achieving a CCC of 0.9902 and an ICC of 0.9773 on sagittal CT images.

**Discussion:** In summary, our method can provide a fully automated measurement of the exophthalmos based on orbital CT images. The proposed method is reproducible, shows high accuracy and objectivity, aids in the diagnosis of relevant orbital diseases, and can quantify treatment effects.

## 1 Introduction

The exophthalmos reflects the anterior-posterior position of the eye relative to the orbit (Segni et al., 2002; Ameri and Fenton, 2004) and is associated with various orbital diseases, including Graves’ orbitopathy, orbital tumor, and orbital fracture (Burch and Wartofsky, 1993; Bartalena et al., 2000; Alsuhaibani et al., 2011; Guo et al., 2018; Huh et al., 2020; Huang et al., 2022; Ji et al., 2022). Accurate measurement of exophthalmos can assist in the diagnosis of these related diseases (Segni et al., 2002; Lam et al., 2010) and also quantify the treatment outcome.

Currently, the main clinical methods for measuring exophthalmos can be classified as exophthalmometer and computed tomography (CT) methods. The most widely used method is the Hertel exophthalmometer (Migliori and Gladstone, 1984; Dunsky, 1992), which measures the distance from the lateral orbital rim to the corneal surface in a direction perpendicular to the frontal plane as a quantitative indicator of the degree of exophthalmos (O’Donnell et al., 1999). However, the Hertel exophthalmometer has low inter- and intraobserver reproducibility, which in turn affects the reliability of its results (Frueh et al., 1985; Musch et al., 1985; Dunsky, 1992; Chang et al., 1995; Kim and Choi, 2001; Sleep and Manners, 2002; Ameri and Fenton, 2004). Furthermore, this method is not suitable for subjects with abnormalities, such as severe upper eyelid swelling, ptosis, and hyper-deviated eyes, because it is greatly influenced by facial tissues (Na et al., 2019).

Meanwhile, clinicians using CT scans for diagnosing the degree of exophthalmos measure the relevant distance manually by dragging the mouse after determining physiological structures, such as the outer edge of the orbit and the apex of the anterior surface of the cornea (Nkenke et al., 2003; Bingham et al., 2016; Na et al., 2019). Such a manual method of measuring exophthalmos is not only time-consuming and inefficient but also inevitably subjective to the clinician, resulting in poor reproducibility of the interobserver measurements (Huh et al., 2020). Therefore, an objective, accurate, convenient, and efficient method for measuring exophthalmos is necessary for the timely diagnosis or assessment of treatment outcomes for relevant orbital diseases. The development of image processing and deep learning methods has provided the basis for automatic, objective, efficient, and accurate computer-aided diagnosis, and these methods have been widely applied in a variety of fields—especially in studies related to the diagnosis of ophthalmic diseases (Zhao et al., 2022).

In this paper, we propose an automated method based on image processing and deep learning to measure the vertical distance from the apex of the anterior corneal surface to the lateral orbital rim of both eyes and the longest line of the superior to the inferior orbital rim on the axial and sagittal plane of CT images, respectively. The two distance parameters, related to ocular prominence, can be measured objectively, accurately, and efficiently without relying on the clinician. This method can help clinicians diagnose diseases related to protrusion or depression by measuring the exophthalmos.

## 2 Materials and methods

### 2.1 Data

Ocular CT images were collected from 31 subjects in the horizontal position and 43 subjects in the sagittal position at the Shenzhen Eye Hospital and Shenzhen Overseas Chinese Hospital using a Philips Ingenuity core 129—a Dutch computed tomography machine with a CT scan thickness of 0.625 mm using the soft tissue window. For this study, 79 horizontal CT images and 99 sagittal CT images including the thickest lens were selected by clinicians empirically.

To train a deep learning network model for automatic eye region segmentation, we divided 79 axial CT images from 31 CT sequence images and 99 sagittal CT images from 43 CT sequence images into a training, validation set, and test set in the ratio of 48:8:23 and 48:8:43, respectively. The ratio of the number of eyes in the training set, validation set and test set for axial and sagittal images is 96:16:40 and 96:16:43, respectively.

Two ophthalmology clinician measured the vertical distance of the line from the apex of the anterior surface of the eye to the most protruding point of the orbital rim for 23 images of the axial plane and 43 images of the sagittal plane. A researcher contributed to the annotation of the ground truth using the “polygon selections” and “fill” function of the software ImageJ (National Institutes of Health, Bethesa, MD, United States) to map the mask of the eye region in all CT images for eye region segmentation by U-Net++ networks.

### 2.2 Overall approach

**FIGURE 1:**
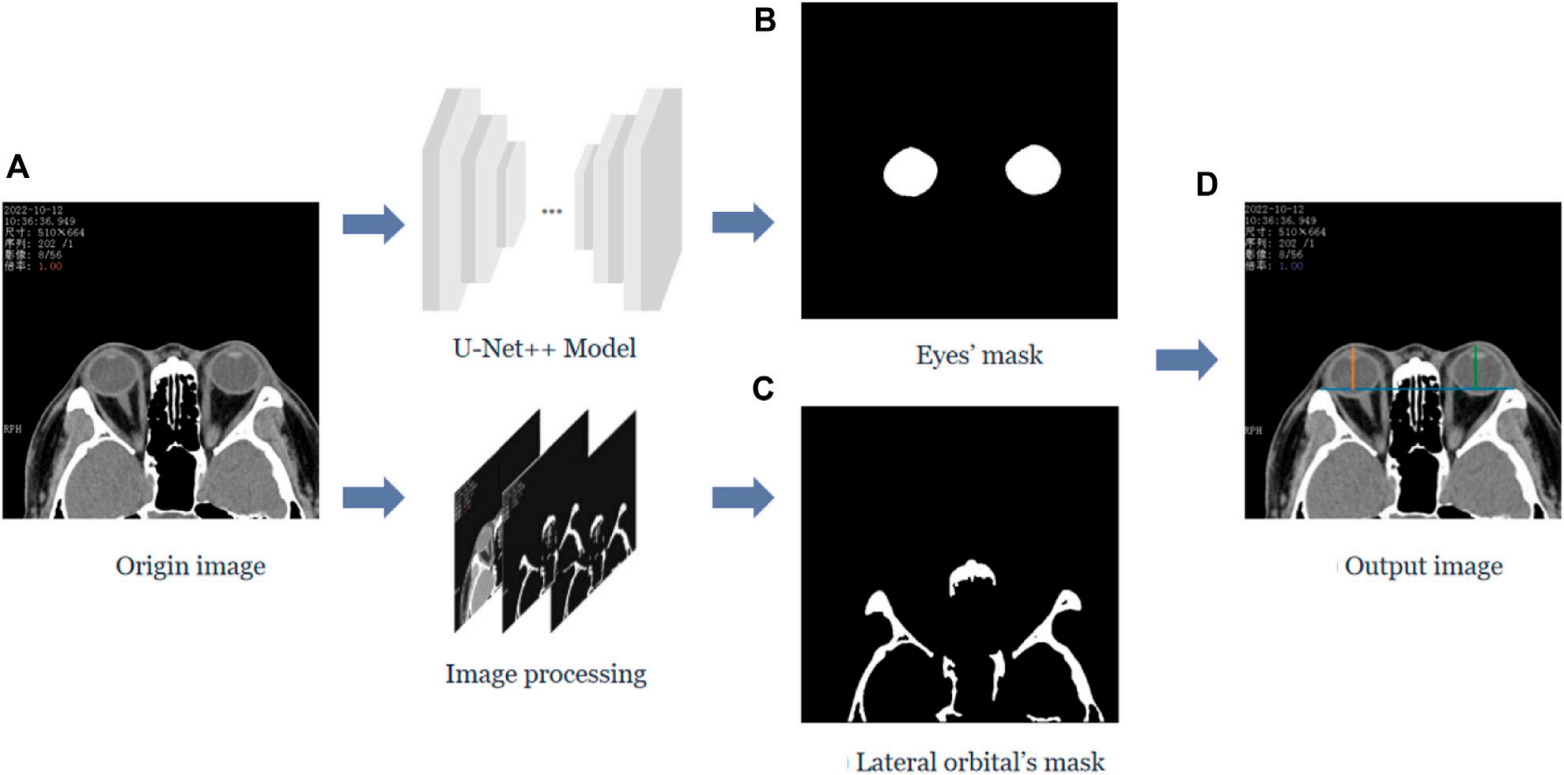
Process of calculating the vertical distance from the apex of the anterior surface of the cornea to the lateral orbital rim of both eyes in the axial plane of the CT image. **(A)** The original axial plane of the CT image, and after the U-Net++ model and Image processing, we can obtain the binary images of the eye region mask shown in **(B)** and the lateral orbital rim region of both eyes shown in **(C)**, respectively. **(D)** The line from the apex of the anterior surface of the eye to the apex of the lateral orbital rim is represented by the blue line in **(D)**, and the vertical distance from the apex of the anterior surface of the eye to the apex of the lateral orbital rim represented by the orange and green lines.

**FIGURE 2:**
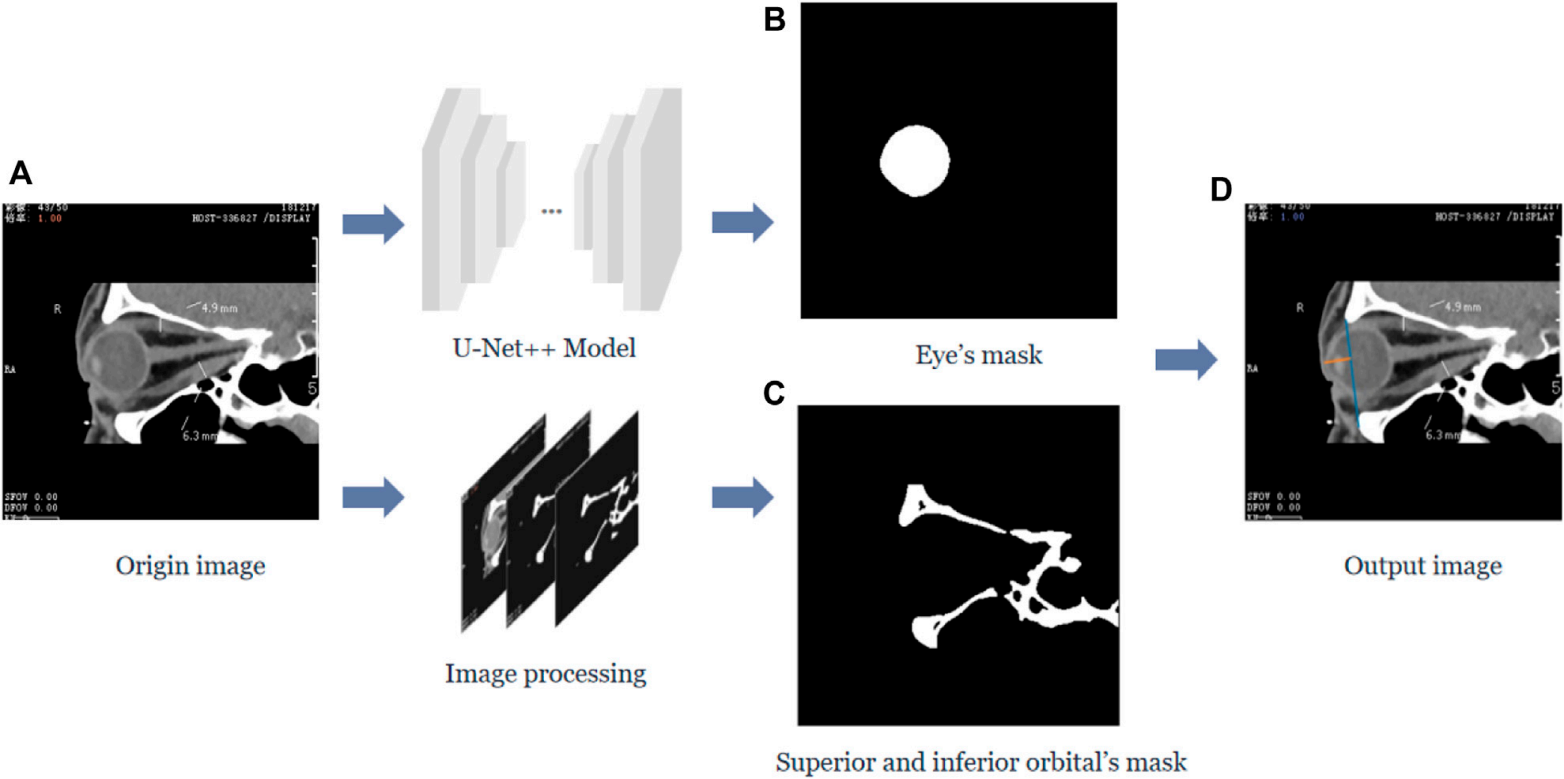
The vertical distance from the apex of the anterior surface of the cornea to the longest line between the superior and inferior orbital margins in the sagittal plane of the CT image. **(A)** Represents the original sagittal CT image, and after the U-Net++ model and Image processing, we obtain the binary images of the eye region mask shown in **(B)** and the upper and lower orbital rim regions shown in **(C)**, and then after locating the coordinates of the anterior surface apex of the eye and the upper and lower orbital rims, we obtain the line from the apex of the upper and lower orbital rims represented by the blue line in **(D)** and the vertical distance of the line from the apex of the anterior surface of the cornea to the upper and lower orbital rim most protruding points represented by the orange line.

In this study, we calculated the vertical distance from the apex of the anterior corneal surface to the lateral orbital rim of both eyes on the axial plane of the CT images, as shown in Figure 1. First, the neural network was trained based on the U-Net++ model for segmenting the eye region in the axial plane of the CT images, and then input the remaining CT images not used for training the model into the segmentation model to obtain a mask of the eye. Furthermore, the lateral orbital rim region of both eyes was obtained after a series of image processing steps. Next, the coordinates of the apex of the anterior corneal surface and the most protruding point of the lateral orbital rim of both eyes were extracted. Additionally, the vertical distance of the line from the apex of the anterior corneal surface to the most protruding point of the upper and lower orbital rim was calculated, as shown in Figure 2.

Similarly, the neural network must be trained to segment the eye region based on the U-Net++ model in the sagittal plane of the CT images while processing the CT images to obtain the upper and lower orbital rim regions. After extracting the coordinates of the apex of the anterior corneal surface and the most protruding point of the upper and lower orbital rim, the vertical distance of the line from the apex of the anterior corneal surface to the most protruding point of the upper and lower orbital rim was calculated.

### 2.3 Segmentation

In order to obtain the best segmentation results, we trained the models commonly used segmentation networks in clinical practice, including FCN32 (Long et al., 2015), SegNet (Badrinarayanan et al., 2017), U-Net (Ronneberger et al., 2015), U-Net++ (Zhou et al., 2018), and Res-U-Net (Zhang et al., 2018), respectively, using the dataset of this paper. The U-Net++ model that achieved the best segmentation performance on the test set was selected for the eye region segmentation.

We implemented the U-Net++ model for eye region segmentation. First, the learning rate and decay rate were set to 0.0001 and 0.99, respectively, Kaiming initialization (He et al., 2015) was used for initializing the weights of the model. Dice loss (Milletari et al., 2016) was used to compare the model segmentation results with the ground truth, and the Adam optimization method (Kingma and Ba, 2014) was applied to minimize the loss value of the network. We inputted the original images and ground truth masks from the training and validation sets to the U-Net++ model. After 200 training iterations of epochs on a server configured with the GPU NVIDIA GeForce GTX 3090TI and using the Pytorch (Paszke et al., 2017) framework, we obtained a network model for automatic eye region segmentation. The formula for Dice loss is shown below.

$$Dice\;loss=1-\left(2*\left|X\cap Y\right|\right)/\left(\left|X\right|+\left|Y\right|\right)$$

*X* represents the eye area mask produced by the neural network segmentation, and Y represents the ground truth of the eye area mask input to the neural network.

In addition to the orbital region, we must segment the lateral orbital rim regions of both eyes in the axial plane as well as the superior and inferior orbital rim regions in the sagittal plane of the CT images to extract the coordinates of their most protruding points. The orbital rim, i.e., the human orbital bone, shows strong contrast in CT images compared to other tissues, so we can use traditional image processing methods to extract the skeletal region.

**FIGURE 3:**
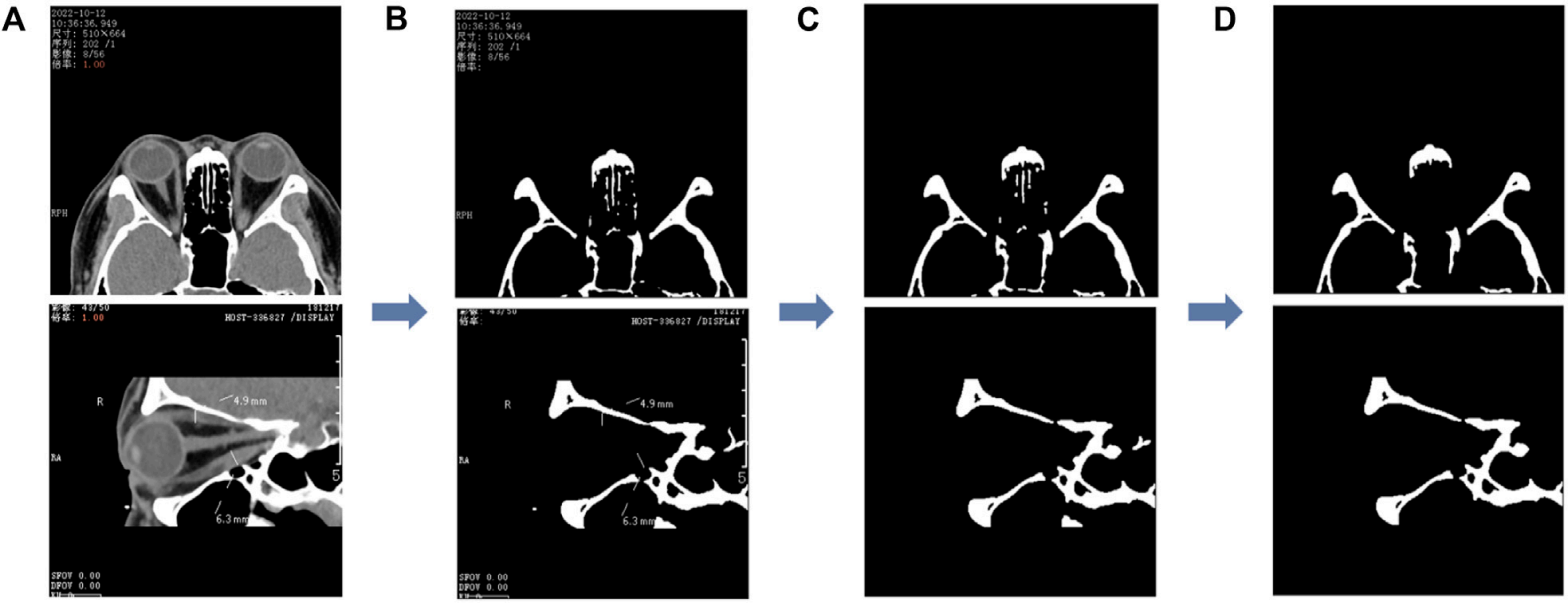
Image processing of the segmented orbital rim region. **(A)** The unprocessed CT image and the binary image in **(B)** are obtained after the threshold segmentation; the binary image shown in **(C)** is obtained after the morphological opening operation, and finally, the smaller connected domain is eliminated to obtain the binary image containing the outer edge of the orbit shown in **(D)**. The first row represents the axial CT image and the second row the sagittal CT image.

We first performed threshold segmentation separately with a grayscale values’ threshold of 200 for the CT images of the two planar views (as in Figure 3A) empirically. After extracting the structures with grayscale values greater than 200 (as in Figure 3B), we eliminated the residual watermark in the CT images, following threshold segmentation, by performing the morphological opening (as in Figure 3C). Finally, after eliminating the smaller connected domains in the images (considered to be noisy), a binary image containing the lateral orbital rims or the upper and lower orbital rims of both eyes was obtained (as in Figure 3D).

### 2.4 Distance calculation

**FIGURE 4:**
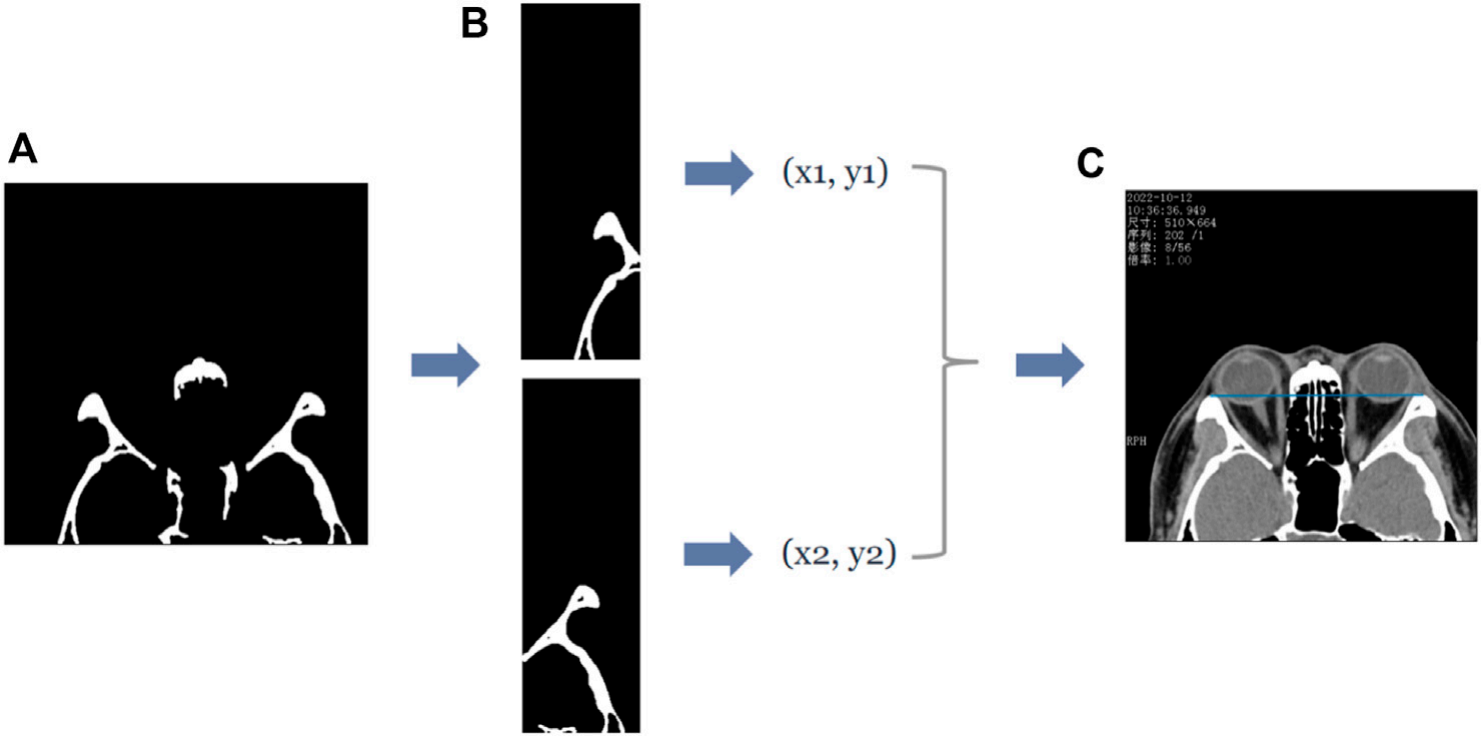
The process of obtaining the coordinates of the most protruding point of the lateral orbital rim of both eyes in the axial plane of the CT image. **(A)** First row of Panel **(B)**, which is the binary image containing the lateral orbital rim region of both eyes. Subsequently, the coordinates of the most protruding points (x1, y1), (x2, y2) of the left and right lateral orbital rims can be determined by traversing the two subgraphs separately, and the straight line passing through the two most protruding points can be visualized by the blue line in **(C)**.

In the axial plane of the CT images, the lateral orbital rims of both eyes were located in the leftmost third of the CT image and the rightmost third of the CT image. Furthermore, the most protruding point can be regarded as the pixel point closest to the top, i.e., the pixel point with the smallest *y*-value in the image coordinate. Therefore, to extract the coordinates of the most protruding point of the lateral orbital rim of both eyes in the axial plane of the CT image, we divided the (d) image in Figure 4 into three subplots: left, middle, and right. First, the images were divided according to the direction of the *x*-axis in the image coordinates, and then the pixel points were traversed in the left and right images in turn. The pixel point with the smallest *y*-value in the “white” area of the two subplots was shortlisted as the coordinate of the most protruding point of the lateral orbital rim of both eyes, as shown in Figure 4. The entire process is shown in Figure 4.

**FIGURE 5:**
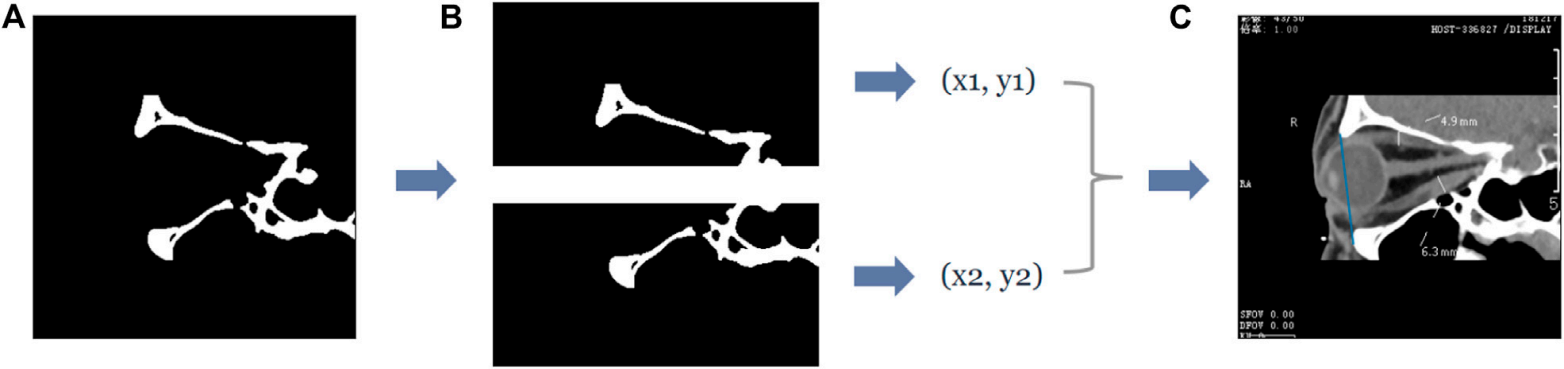
The process of obtaining the coordinates of the most protruding points of the upper and lower orbital rim in the sagittal plane of the CT image. **(A)** The second row of Figure 4B, which is the binary image containing the upper and lower orbital rim regions, and extracts the submaps of the upper and lower halves, respectively, to obtain the two submaps as shown in **(B)**. Subsequently, the coordinates of the most protruding points of the superior and inferior orbital rims can be determined by traversing the two subgraphs separately, and then the straight line passing through the two most protruding points can be obtained—the straight line is shown in blue in **(C)**.

In the sagittal plane of the CT images, the upper and lower orbital margins are located in the upper and lower molecular maps of the CT image, respectively—their most protruding point can be regarded as the point closest to the left side of the image, i.e., the pixel point with the smallest *x*-value in the image coordinates. Therefore, we follow an operation similar to that of the axial plane–the pixel points in the “white” area in the upper and lower submaps are traversed, respectively, and the point with the smallest *x*-value is recorded as the coordinate of the most protruding point of the upper and lower orbital margins. The entire process is shown in Figure 5.

After obtaining the coordinates of the lateral orbital rims of both eyes, we obtained the equation of the line passing through the two points of the lateral orbital rims of both eyes. Similarly, we obtained the equation of the line passing through the two points of the upper and lower orbital rims based on the coordinates of the most protruding points of the upper and lower orbital rims in the sagittal plane of the CT image. Subsequently, we traversed the pixel points of the eye region mask output by the eye region segmentation model and recorded the point with the smallest *x*-value among the mask pixel points as the coordinates of the most protruding point of the anterior corneal surface vertex. From this, we calculated the vertical distance from the apex of the anterior corneal surface to the lateral orbital rim of both eyes in the axial plane of the CT image and the vertical distance from the apex of the anterior corneal surface to the upper and lower orbital rims in the sagittal plane of the CT image. The results of our automated method and the manual measurements by the physicians were compared.

### 2.5 Statistical analysis

The Dice coefficient (Dice, 1945), Intersection Over Union (IOU), precision, and recall were used as metrics to evaluate the segmentation performance of the model. The metrics were calculated as shown below.

$$Dice=2TP/\left(FP+2TP+FN\right)$$

$$IOU=TP/\left(TP+FN+FP\right)$$

$$Precision=TP/\left(TP+FP\right)$$

$$Recall=TP/\left(TP+FN\right)$$

The intraclass correlation coefficient (ICC) and the concordance correlation coefficient (CCC) were used to demonstrate the concordance between the results of our automated method and the manual measurements of the physicians. The two-way mixture model and the absolute consistency type were chosen for the calculation of the intra-group correlation coefficients.

## 3 Results

### 3.1 Ocular segmentation

**FIGURE 6:**
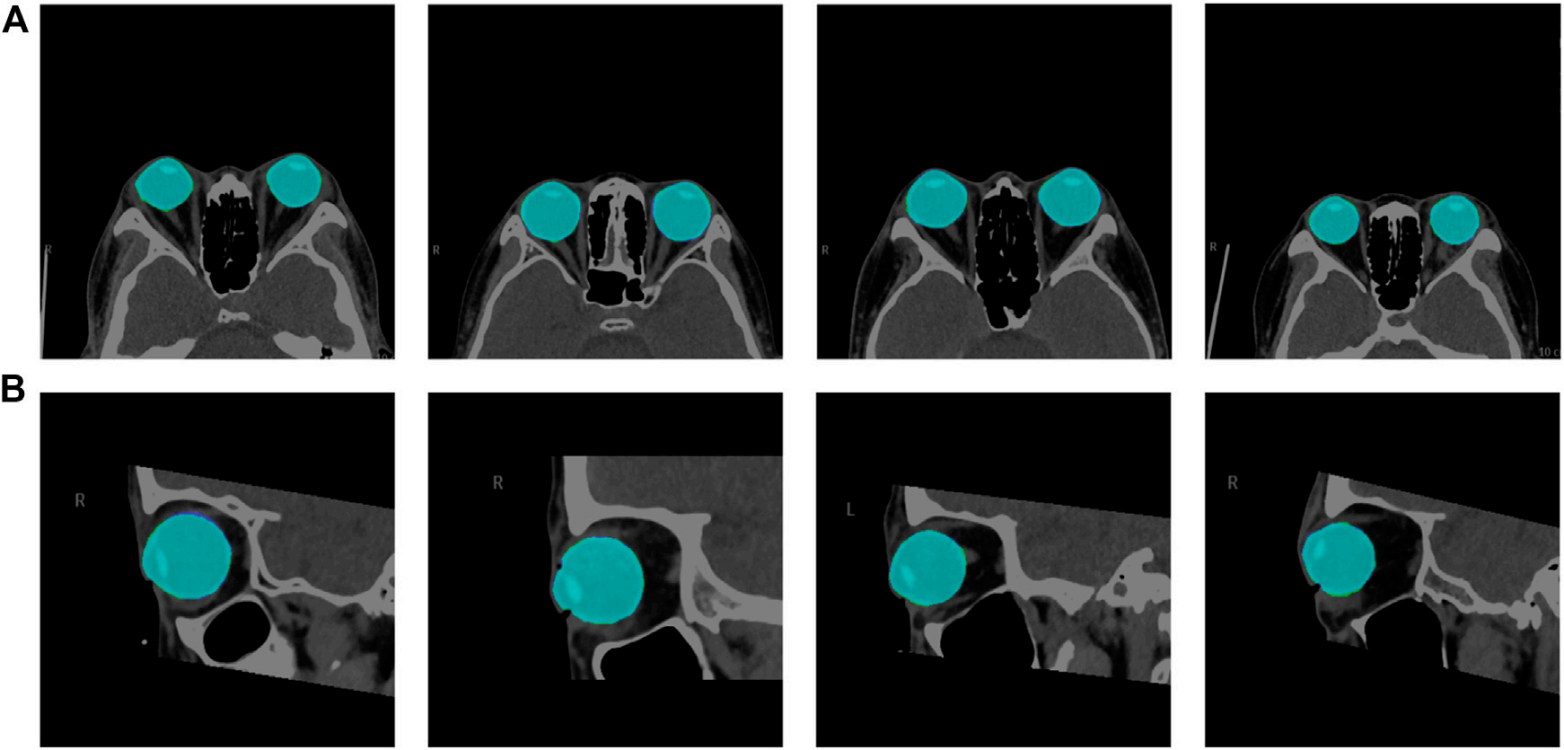
Visualization segmentation results of the model used to segment the eye region. Row **(A)** represents the visual segmentation results of the model in the axial plane of the test set, and row **(B)** represents the visual segmentation results of the model in the sagittal plane of the test set. The purple and green area represent the segmentation masks predicted by the manual and network, respectively. The light blue area is the overlapping part of both, i.e., the correctly predicted eye area.

We used the segmentation results of 40 eyes in 23 horizontal CT images and 43 eyes in 43 sagittal CT images to test the ability of the model to segment eye region. Table 1 shows the segmentation performance of the five models on the test set data, and Figure 6 shows the visualization of the U-Net++ model segmentation results. From the results, we observe that the region of the eye was segmented accurately in both horizontal and sagittal CT images. This indicates that this method can accurately locate the vertex coordinates of the anterior surface of the eye.

**TABLE 1:** The mean values (standard deviation) of the Dice Coefficient, IOU, Precision, Recall for segmenting the ocular region model in the axial plane and the sagittal plane of the CT images, respectively.

View	Model	Dice	Recall	Precision	IOU
The axial plane	FCN32	0.8847 (0.0150)	0.8213 (0.0731)	0.9660 (0.0380)	0.7951 (0.0572)
	SegNet	0.9684 (0.0163)	0.9802 (0.0118)	0.9577 (0.0329)	0.9382 (0.0295)
	U-Net	0.9757 (0.0150)	0.9529 (0.0271)	0.9887 (0.0086)	0.9636 (0.0302)
	U-Net++	0.9805 (0.0059)	0.9829 (0.0090)	0.9782 (0.0140)	0.9617 (0.0112)
	Res-U-Net	0.9805 (0.0073)	0.9838 (0.0085)	0.9775 (0.0164)	0.9619 (0.0138)
The sagittal plane	FCN32	0.8425 (0.0587)	0.7487 (0.0878)	0.9729 (0.0335)	0.7319 (0.0813)
	SegNet	0.9570 (0.0376)	0.9506 (0.0517)	0.9662 (0.0463)	0.9200 (0.0650)
	U-Net	0.9770 (0.0135)	0.9804 (0.0170)	0.9740 (0.0224)	0.9553 (0.0248)
	U-Net++	0.9816 (0.0087)	0.9802 (0.0158)	0.9831 (0.0087)	0.9640 (0.0164)
	Res-U-Net	0.9505 (0.0809)	0.9273 (0.1201)	0.9849 (0.0299)	0.9150 (0.1223)

### 3.2 Ocular prominence measurement

**FIGURE 7:**
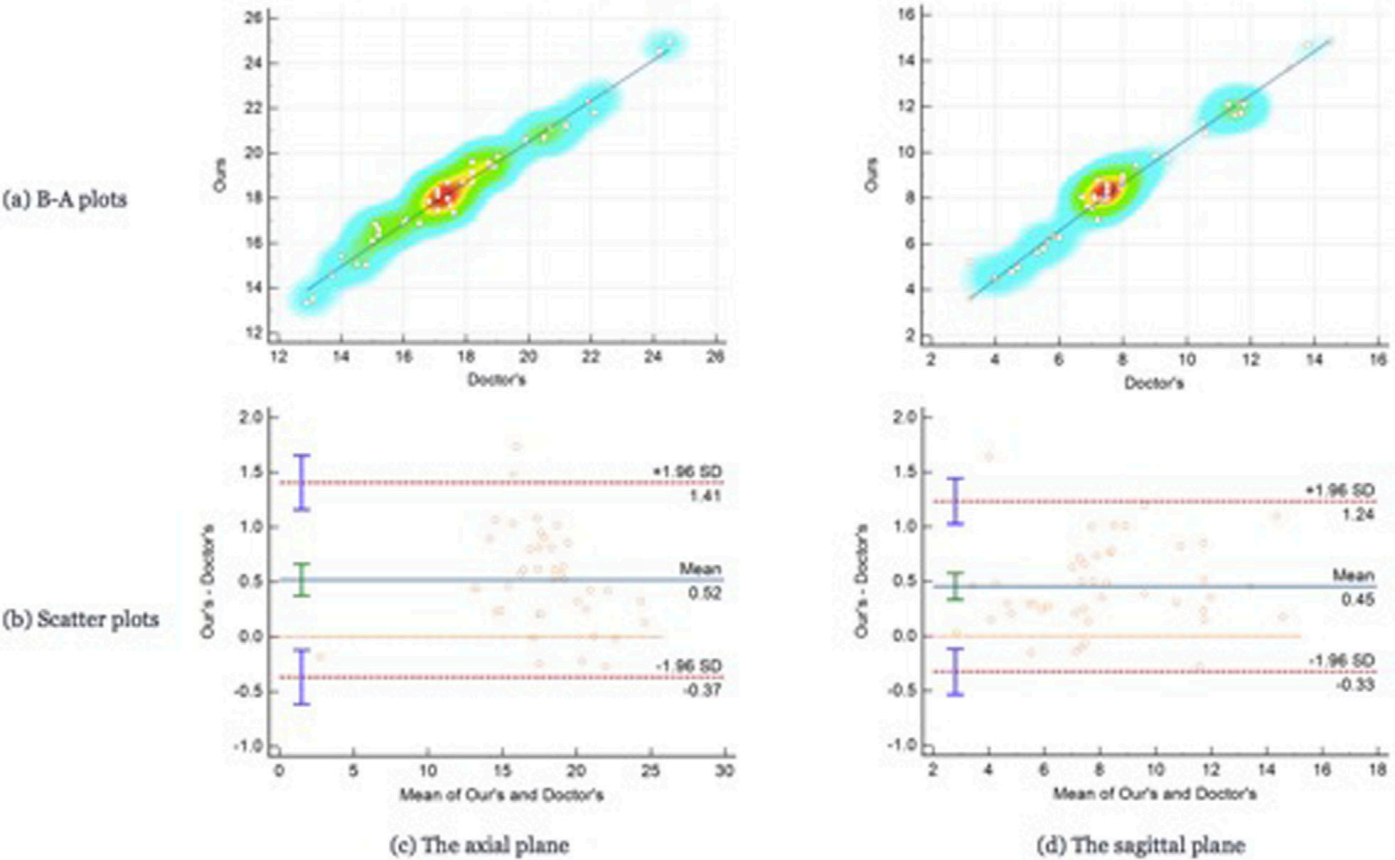
The Bland-Altman plots and the scatter plots between the results on the test set using the automated method proposed in this paper. Row **(A)** represents the Bland-Altman plots and row **(B)** is the scatter plot, column **(C)** means the results on the axial plane while column **(D)** is the results on the sagittal plane. In the scatter plots, the *y*-axis represents the results calculated by the automated method we proposed in this paper while the *x*-axis means the results measured by the doctors.

**FIGURE 8:**
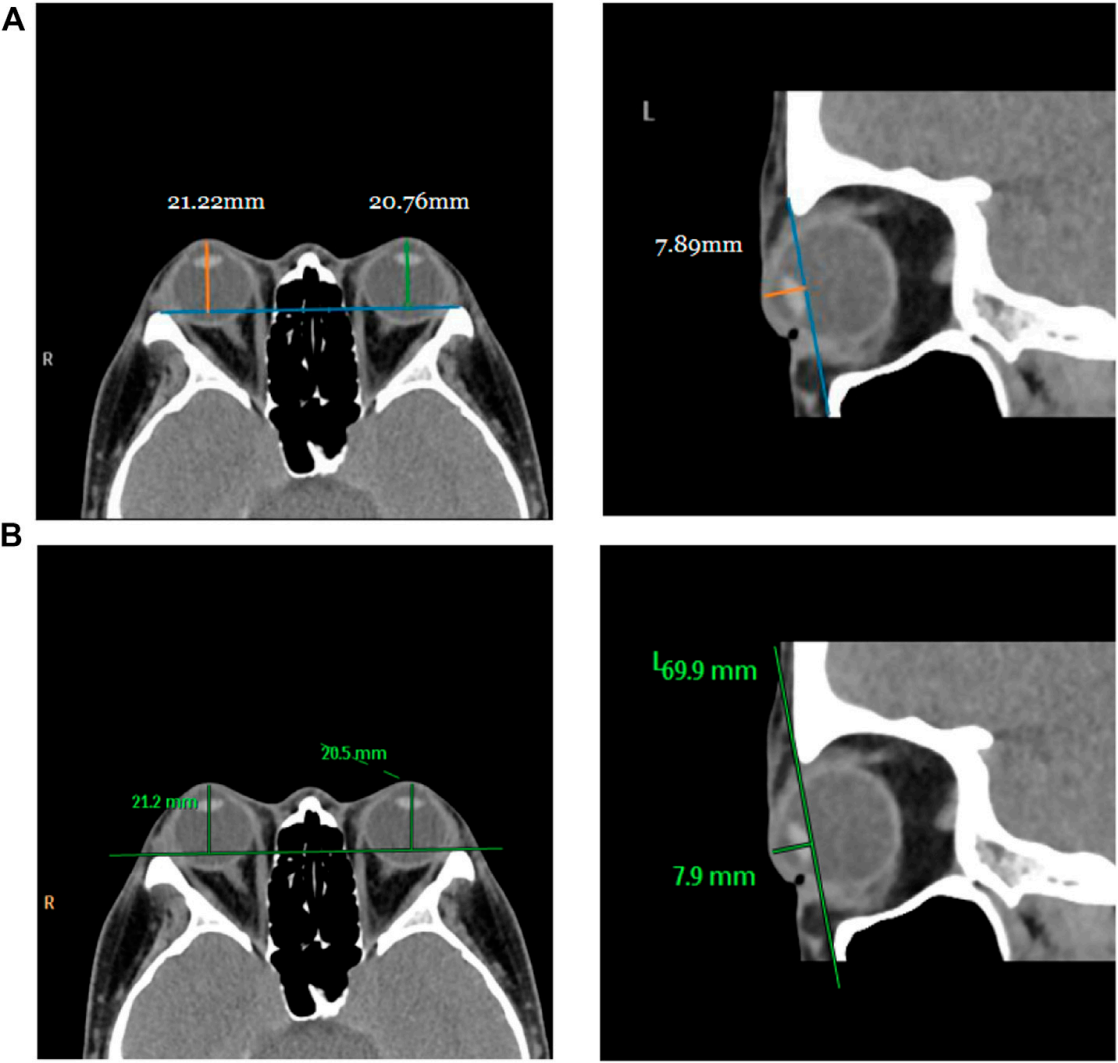
Visualization of measurement results of the proposed automated method and the manual measurement method by the physician on the test set. Row **(A)** represents the visualization result of measurement using the proposed method, and row **(B)** is the visualization result of manual measurement by the physician on the same image.

For testing, we used the vertical distance from the apex of the anterior corneal surface to the most protruding point of the lateral orbital rim of both eyes in 40 eyes from 23 CT images in the axial plane and the vertical distance from the apex of the anterior corneal surface to the most protruding point of the upper and lower orbital rims in 43 eyes from 43 CT images in the sagittal plane. Table 2 shows the mean values (standard deviation) of the Exophthalmometric values between the results measured by the proposed automated method and the manually measured results. The Bland-Altman plots and the scatter diagram between the computed results of the proposed automated method of ocular prominence measurement on the test set and its corresponding manual measurement by the physician is shown in Figure 7. Figure 8 shows the results of the proposed automated method and the manual measurement by the physician on an axial plane and one sagittal plane of the CT image, respectively. The results of the statistical analysis between the measurements using the two methods on all test sets are shown in Table 3.

**TABLE 2:** The mean values (standard deviation) of the Exophthalmometric values between the results measured by the proposed automated method and the manually measured results.

View	Manual method	Automated method
The axial plane	17.83 (2.85)	18.37 (2.67)
The sagittal plane	8.01 (2.79)	8.48 (2.82)

**TABLE 3:** Results of statistical analysis of the concordance correlation coefficient (CCC) and the intraclass correlation coefficient (ICC) between the results measured by the proposed automated method and the manually measured results.

View	CCC	ICC
The axial plane	0.9895	0.9698
The sagittal plane	0.9902	0.9773

From the results of the statistical analysis, it can be observed that although the Bland-Altman plots diagram as well as the mean values of the Exophthalmometric values show a stable error in the results measured by the proposed automated method and the manually measured results, our method is in good agreement with the results of the manual measurement by the physician for both vertical distances. Thus, the accuracy of this method can be verified.

## 4 Discussion

**FIGURE 9:**
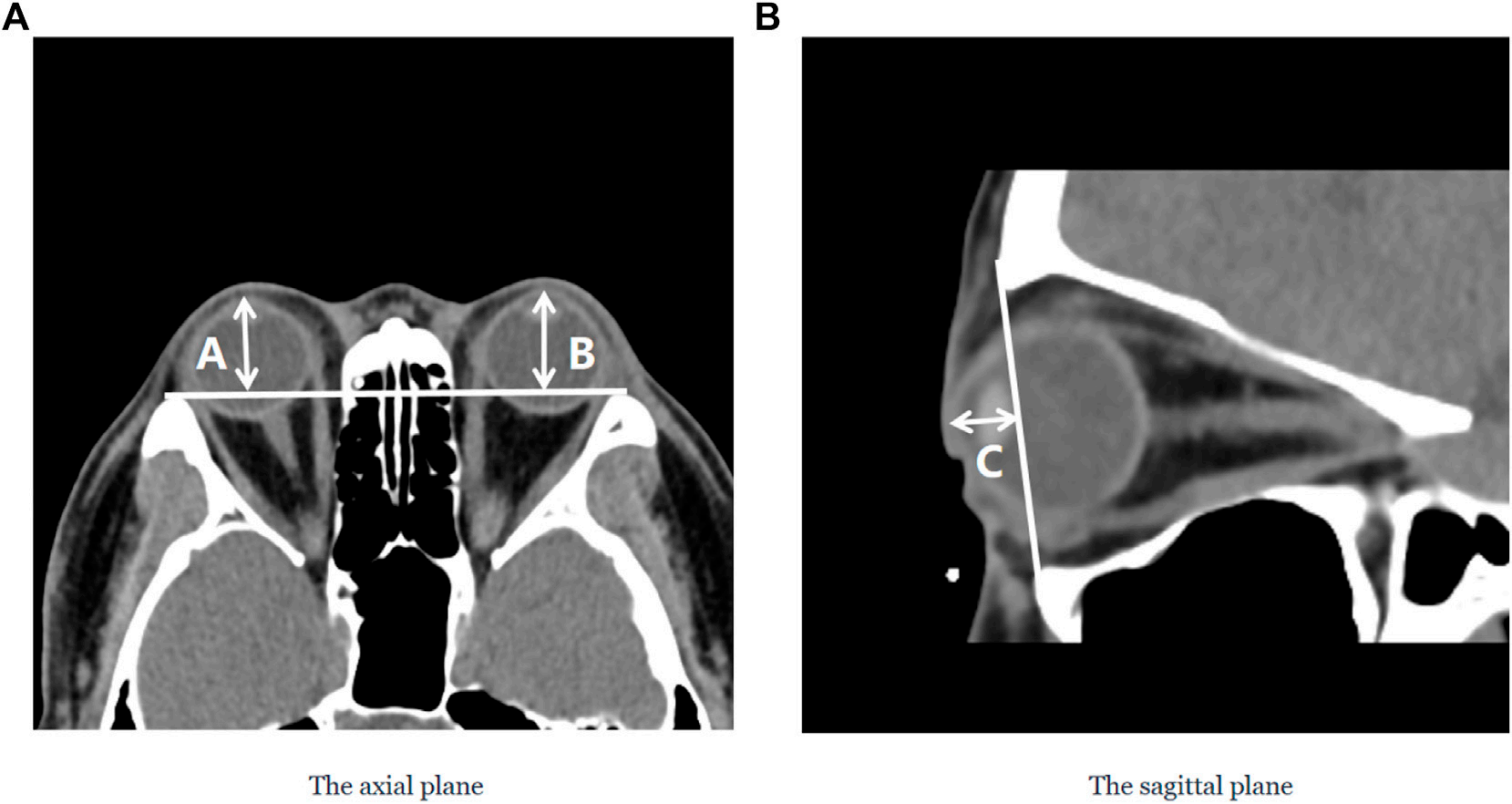
Two commonly used clinical parameters for measuring the prominence of the eye based on CT images. **(A)** The axial plane of the CT image, (A and B) represent the vertical distance from the apex of the anterior corneal surface to the lateral orbital rim of both eyes, respectively; **(B)** The sagittal plane of the CT image, C represents the vertical distance from the apex of the anterior corneal surface to the longest line of the upper and lower orbital rims.

The degree of orbital protrusion is associated with a variety of orbital diseases, and its accurate quantification is important to diagnose certain orbital diseases and determine the effectiveness of their treatment. CT imaging has been used to measure the prominence of the eye because of its high-resolution accuracy and ability to analyze multiple views simultaneously (Kim and Choi, 2001; Nkenke et al., 2003; 2004; Fang et al., 2013). Some studies have shown that CT image-based ocular prominence measurements are more accurate (Hallin and Feldon, 1988; Segni et al., 2002; Nkenke et al., 2003; 2004; Ramli et al., 2015) and correlate well with measurements using the Hertel ocular prominence meter (Klingenstein et al., 2022). For special subjects such as children and those suffering from ptosis, using CT images to measure ocular prominence is the only feasible method. Currently, the most common clinical method is to manually measure the vertical distance from the apex of the anterior corneal surface to the lateral orbital rim of both eyes on an axial CT image as a measure of ocular prominence (as shown in Figure 9A ) (“Axial Globe Position Measurement: A Prospective Multicenter Study by the International Thyroid Eye Disease Society,” 2016; Nkenke et al., 2004). However, when the subject’s head is tilted, using only one plane of view may lead to large errors. In 2019, Na et al. (Na et al., 2019) proposed a method to represent ocular prominence in sagittal CT images by measuring the vertical distance of the longest line connecting the anterior surface apex of the cornea to the superior orbital rim to the inferior orbital rim (as shown in Figure 9B). The method proposed by Park et al. has been validated to be comparable to the Hertel exophthalmometer method with high correlation while being applicable to subjects with horizontal and vertical strabismus. Therefore, a exophthalmos measuring method that combines the two planar views described above would be applicable to a wider population with guaranteed accuracy.

In this paper, we propose a method based on deep learning and image processing techniques to combine axial and sagittal CT images for the automatic measurement of exophthalmos. The experimental results show that our method can achieve accurate segmentation results with Dice coefficients of 0.976 ± 0.015 and 0.977 ± 0.0135 for the eye region in the axial and sagittal plane of the CT images, respectively, on the dataset used in this paper, as shown in Figure 6. We used image processing techniques to segment the orbital region to achieve accurate localization of the apex of the anterior surface of the eye and the most protruding point of the outer edge of the orbit. Based on the results obtained, the CCC and ICC between the two methods were 0.988 and 0.957 for the axial plane of the CT images, respectively, and 0.990 and 0.965 for the sagittal plane of the CT images, respectively—in our dataset of 23 axial and 43 sagittal CT images, which shows high consistency.

The deep learning and digital image processing methods used in our study can automatically segment the structures of the eye and orbital rim, and locate the apex of the anterior corneal surface and the most protruding point of the orbital rim. The process can then calculate the relevant parameters, ensuring the high accuracy and reproducibility of this method to a certain extent in our dataset. Furthermore, the suggested approach can determine the relevant exophthalmos measurements in both axial and sagittal planes of CT scans, offering medical professionals a multi-dimensional reference for diagnosing orbital disorders in patients displaying abnormal exophthalmos seen only in the axial or sagittal plane. After conducting a PubMed search using the keywords “proptosis” and “CT,” we discovered 57 relevant studies published in the past 20 years. However, all of these studies relied on manual drawings and measurements performed by clinicians or researchers, which can be remedied by implementing the proposed method. Additionally, the full automation of the process in this paper not only minimizes the impact of subjective factors on measurement results, but also enhances measurement efficiency. On average, the time required to calculate the vertical distance from the anterior corneal surface’s apex to the most protruding point of the lateral orbital rim in axial CT images and the vertical distance from the anterior corneal surface’s apex to the most protruding points of the upper and lower orbital rims in sagittal CT images is 0.9 s and 0.75 s, respectively. This automated method significantly reduces the time and effort required for eye protrusion measurement compared to manual methods.

The work in this paper was performed on 2D CT images, which meets the practical needs of current clinicians for diagnosis (Kim and Choi, 2001), especially in patients with eyelid exophthalmos and other conditions (Na et al., 2019). It is worth mentioning that some research teams have implemented the quantification of ocular prominence on 3D CT images (Guo et al., 2017; 2018; Huh et al., 2020; Willaert et al., 2020), but none of them have been fully automated. However, the mainstream methods in clinical practice are still dominated by the Hertel ocular prominence meter method and the lightweight-based 2D CT image method. The 3D CT image-based ocular prominence measurement method is complex and time-consuming, and we will explore other ocular prominence-related parameters (Kim and Choi, 2001; Campi et al., 2013; Guo et al., 2017; Afanasyeva et al., 2018; Choi and Lee, 2018) in our future work, for automatic measurement and validate their practical feasibility in a clinical setting.

However, the approach in this paper applies to both axial and sagittal CT images and requires the most protruding point of the outer edge of the orbit to determine the measurement of the exophthalmos, which is a limitation in case of some images with incomplete, missing or displaced outer orbit edges.

## 5 Conclusion

This study introduces an automated approach for assessing eye prominence in both axial and sagittal CT images of the orbit using deep learning and image processing techniques. This method eliminates the need for prior knowledge from clinicians, thereby reducing their workload. On the experimental dataset, the method shows satisfactory efficiency, accuracy, reliability, and reproducibility. This approach has the potential to support the diagnosis and treatment quantification of related orbital diseases.

## Data Availability

The raw data supporting the conclusion of this article will be made available by the authors, without undue reservation.
